# The PRDM9 KRAB domain is required for meiosis and involved in protein interactions

**DOI:** 10.1007/s00412-017-0631-z

**Published:** 2017-05-19

**Authors:** Yukiko Imai, Frédéric Baudat, Miguel Taillepierre, Marcello Stanzione, Attila Toth, Bernard de Massy

**Affiliations:** 1 Institut de Génétique Humaine UMR9002 CNRS-Université de Montpellier, 141 rue de la cardonille, 34396 Montpellier cedex 05, France; 2 SEAT-TAAM CNRS Phenomin UPS44, 7 rue Guy Môquet, 94800 Villejuif, France; 3 Faculty of Medicine at the TU Dresden, Institute of Physiological Chemistry, Fetscherstraße 74, 01307 Dresden, Germany

**Keywords:** PRDM9 KRAB domain, Meiosis, Protein interactions, Recombination, CXXC1, IHO1

## Abstract

PR domain-containing protein 9 (PRDM9) is a major regulator of the localization of meiotic recombination hotspots in the human and mouse genomes. This role involves its DNA-binding domain, which is composed of a tandem array of zinc fingers, and PRDM9-dependent trimethylation of histone H3 at lysine 4. PRDM9 is a member of the PRDM family of transcription regulators, but unlike other family members, it contains a Krüppel-associated box (KRAB)-related domain that is predicted to be a potential protein interaction domain. Here, we show that truncation of the KRAB domain of mouse PRDM9 leads to loss of PRDM9 function and altered meiotic prophase and gametogenesis. In addition, we identified proteins that interact with the KRAB domain of PRDM9 in yeast two-hybrid assay screens, particularly CXXC1, a member of the COMPASS complex. We also show that CXXC1 interacts with IHO1, an essential component of the meiotic double-strand break (DSB) machinery. As CXXC1 is orthologous to *Saccharomyces cerevisiae* Spp1 that links DSB sites to the DSB machinery on the chromosome axis, we propose that these molecular interactions involved in the regulation of meiotic DSB formation are conserved in mouse meiosis.

**Electronic supplementary material:**

The online version of this article (doi:10.1007/s00412-017-0631-z) contains supplementary material, which is available to authorized users.

## Introduction

Meiotic recombination is initiated by programmed DNA double-strand breaks (DSBs), generated by SPO11 and accessory proteins (de Massy 2013). Meiotic DSBs are not randomly distributed within the genome but mostly occur at discrete regions, called hotspots (Baudat et al. 2013). Interestingly, the molecular mechanisms underlying the patterning of meiotic DSBs are quite distinct in different species with apparently two major pathways. One pathway, which has been extensively studied in yeast, is mainly guided by chromatin structure and the other, analyzed in detail in mice, is driven by the sequence-specific DNA-binding PR domain-containing protein 9 (PRDM9).

In *Saccharomyces cerevisiae*, meiotic DSBs preferentially occur at nucleosome-depleted regions in gene promoters (Ohta et al. 1994; Pan et al. 2011; Wu and Lichten 1994), where the level of trimethylation of histone H3 at lysine 4 (H3K4me3) is constitutively high (Borde et al. 2009). At a larger scale, DSB formation occurs in the context of a specific chromosome architecture that consists of chromatin loops anchored to a proteinaceous axis (Zickler and Kleckner 1999). DSB sites are preferentially located within chromatin loops, while several proteins that are required for DSB formation (Rec114, Mei4, and Mer2) localize on the chromosome axis (Blat et al. 2002; Panizza et al. 2011). Thus, DSB sites should interact with the chromosome axis before or at the time of DSB formation. In *S. cerevisiae*, this interaction is at least in part provided by Spp1 that directly interacts with both methylated H3K4 near DSB sites and the axis-localized protein Mer2 (Acquaviva et al. 2013; Sommermeyer et al. 2013).

In humans and mice, hotspots are specified by PRDM9, a meiosis-specific PRDM family protein (Baudat et al. 2010; Myers et al. 2010; Parvanov et al. 2010). PRDM9 includes several functional domains, such as a DNA-binding zinc finger array, a histone lysine methyltransferase PR/SET (PRDI-BF1 and RIZ1 homology) domain, and a Krüppel-associated box (KRAB)-related domain. The PRDM9 zinc finger array is highly polymorphic both within and between species (Berg et al. 2010; Buard et al. 2014; Groeneveld et al. 2012; Kono et al. 2014; Oliver et al. 2009; Thomas et al. 2009). Strikingly, two congenic mouse strains in which the *Prdm9* alleles have different zinc finger arrays show completely distinct sets of hotspots (Brick et al. 2012), generalizing the observation that hotspot localization is determined by the DNA-binding specificity of the zinc finger array (Grey et al. 2011). The PR/SET domain of PRDM9 catalyzes H3K4me3 in vitro (Eram et al. 2014; Hayashi et al. 2005; Koh-Stenta et al. 2014; Wu et al. 2013), and this post-translational modification is enriched at DSB hotspots in a PRDM9-dependent manner (Baker et al. 2014; Brick et al. 2012; Grey et al. 2011). However, H3K4me3 is also enriched at promoters, but these sites are not used for meiotic DSB formation (Pratto et al. 2014; Smagulova et al. 2011). This implies that H3K4me3 is not sufficient for DSB site specification when PRDM9 is present. Indeed, in the absence of PRDM9, meiotic DSBs occur at transcription regulatory regions, such as promoters and enhancers (Brick et al. 2012). Thus, although the requirement of H3K4me3 for DSB formation remains to be determined in mice, its presence near DSB sites, when PRDM9 is present or absent, is reminiscent of the DSB features described in *S. cerevisiae*. In the mouse, MEI4 and IHO1, two essential components for meiotic DSB formation, localize on unsynapsed chromosome axes where they are detected at the beginning of meiotic prophase when DSB formation takes place (Kumar et al. 2010; Stanzione et al. 2016). In addition, HORMAD1, a component of the chromosome axis that interacts with IHO1, is required for wild-type DSB levels (Daniel et al. 2011). This suggests that an interaction may be needed to bring PRDM9-bound sites where DSBs form (Lange et al. 2016) into proximity of DSB-promoting complexes on chromosome axes. Indeed, we have detected interactions between PRDM9 and potential axis sites compatible with this hypothesis (Grey et al. 2017) and several axis proteins interact with PRDM9 (Parvanov et al. 2017). Interestingly, PRDM9 harbors a KRAB-related domain that generally mediates protein–protein interactions (Lupo et al. 2013). This KRAB-related domain is present only in PRDM9 and PRDM7 among all PRDM family proteins (Hohenauer and Moore 2012), and its role is unknown. We hypothesized that it could be involved in the direct interaction between PRDM9 and a component (s) that enables the recruitment of hotspots to DSB proteins on the chromosome axis. To test this hypothesis, we generated a deletion in *Prdm9* that leads to a truncation of this domain and showed that the PRDM9 KRAB domain is essential for meiosis progression, meiotic DSB repair, and synapsis in both female and male mice. In addition, we identified proteins that interact with the KRAB domain of mouse and human PRDM9 by yeast two-hybrid (Y2H) screening. The finding that CXXC1, the mammalian orthologue of *S. cerevisiae* Spp1, specifically interacts with the PRDM9 KRAB domain and also with IHO1 leads us to propose a role for PRDM9 KRAB in linking DSB hotspots to the chromosome axis.

## Results and discussion

### The PRDM9 KRAB domain is essential for meiosis

**Fig. 1:**
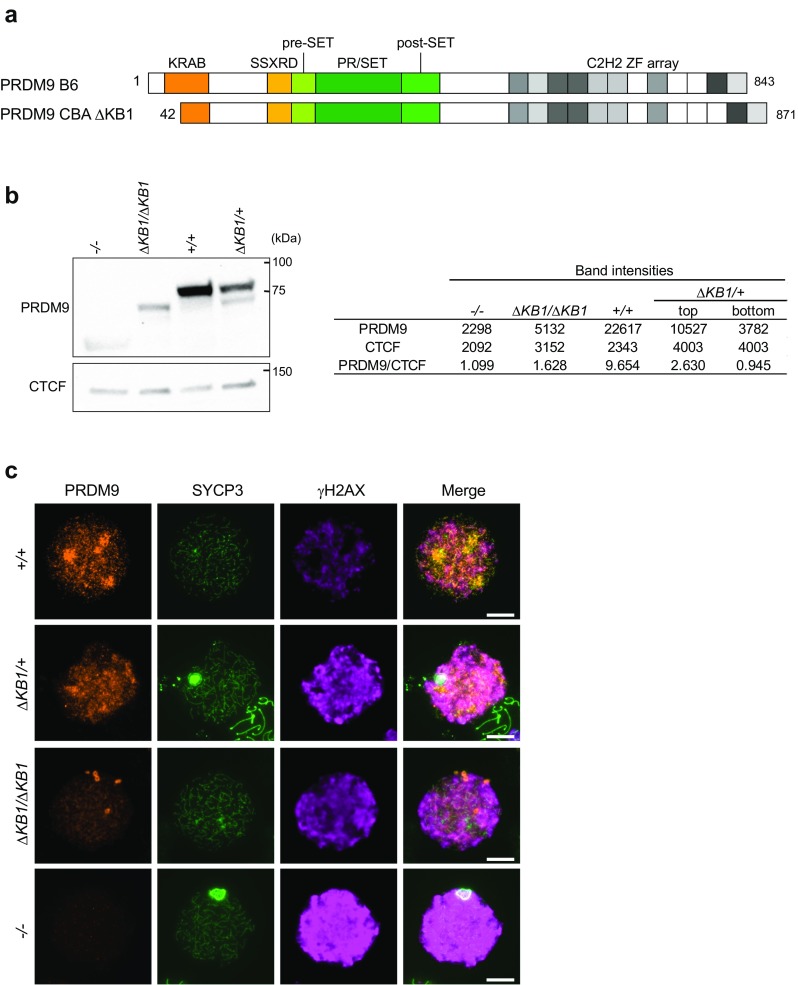
A 26-bp deletion of the *Prdm9* gene leads to production of an N-terminal truncated PRDM9 protein. **a** Schematic representation of the PRDM9 proteins expressed from the wild-type B6 allele and the CBA *ΔKB1* allele of the *Prdm9* gene. These two alleles have distinct zinc finger arrays. **b** Immunoblot of PRDM9 expression in *Prdm9* ^*−/−*^, *Prdm9* ^*ΔKB1/ΔKB1*^, *Prdm9* ^*+/+*^ (B6), and *Prdm9* ^*ΔKB1/+*^(heterozygous *Prdm9* ^*ΔKB1*^/*Prdm9* ^*Dom2*^ from B6) testes at 13 dpp. Each lane was loaded with 100 μg of testis protein extracts. The same membrane was stained with an anti-CTCF antibody as loading control. The predicted molecular weights of PRDM9 B6 and PRDM9ΔKB1 are 97 and 95 kDa, respectively. Both proteins migrate faster than predicted. The *right panel* reports signal intensities in arbitrary units of each band (PRDM9, CTCF and the *top* and *bottom* PRDM9 bands in ΔKB1/+) and the ratio of intensities between PRDM9 and CTCF (PRDM9/CTCF). **c** Immunofluorescent analysis of PRDM9, SYCP3 (meiotic chromosomes axial elements marker), and γH2AX (DSB formation marker) expression in chromosome spreads of *Prdm9* ^*+/+*^ (B6), *Prdm9* ^*ΔKB1/+*^ (heterozygous *Prdm9* ^*ΔKB1*^/*Prdm9* ^*Dom2*^ from B6), *Prdm9* ^*ΔKB1/ΔKB1*^, and *Prdm9* ^*−/−*^ spermatocytes at leptonema. Within one genotype, intensity of γH2AX can vary at leptonema and we did not detect consistent differences between genotypes. Chromosome spreads were prepared from 10-week-old (*Prdm9* ^*+/+*^, *Prdm9* ^*ΔKB1/+*^, and *Prdm9* ^*ΔKB1/ΔKB1*^) or 5-week-old (*Prdm9* ^*−/−*^) mice. *Scale bars* = 10 μm

The PRDM9 KRAB domain includes subdomains A and B and is located between residues 23 and 86 of the protein. It shares strong similarity with the KRAB domain of SSX proteins, and conserved residues are mostly in the KRAB A domain that has a predicted alpha helical structure (Supplementary Fig. 1a) (Birtle and Ponting 2006). Conversely, the sequences of the KRAB domains of PRDM9 and SSX proteins are quite divergent from those of canonical KRAB domain proteins, such as KOX1 (Supplementary Fig. 1a). To determine its function in the mouse, we introduced a deletion in the KRAB domain by injecting Cas9 mRNA and a single-guide RNA that targets the 5′ site of *Prdm9* in C57BL/6(B6) × CBA F1 zygotes (see “Materials and methods”) (Supplementary Fig. 1b, c). We identified a deletion of 26 bp in the CBA allele of *Prdm9* that has an additional zinc finger compared with B6 mice. This deletion led to a frameshift and a predicted protein that is translated from an internal AUG codon and lacks the first 42 amino acids, including several conserved residues of the KRAB A domain (Fig. 1a and Supplementary Fig. 1d). We detected this truncated protein (called PRDM9ΔKB1 hereafter) in mouse testis protein extracts by western blotting (Fig. 1b). We noted that the migration of this PRDM9ΔKB1 protein was faster than predicted since its predicted molecular weight is close to the one of the wild-type B6 PRDM9 control (95 and 97 kDa, respectively). This could suggest that PRDM9ΔKB1 is translated from more internal AUG codons and/or that post-translational modifications alter the migration. Moreover, PRDM9ΔKB1 protein level was lower than that of wild-type PRDM9 (5.9- to 2.9-fold) in both *Prdm9* ^*ΔKB1*/*ΔKB1*^ and *Prdm9* ^*ΔKB1*/*+*^ protein extracts (Fig. 1b, right panel). Similarly, by immunofluorescence analysis in spread spermatocytes, we could observe that PRDM9ΔKB1 was detected in the nucleoplasm but at a lower level, compared to wild-type PRDM9, at leptonema, the stage at which it is most abundant (Grey et al. 2017). The reduction of PRDM9 in *Prdm9* ^*ΔKB1*/*ΔKB1*^ compared to wild type was from 1.3- to 3.4-fold in early or late leptonema depending on whether the signal from *Prdm9* ^*−/−*^ was subtracted or not (Fig. 1c and Supplementary Fig. 2).

**Fig. 2:**
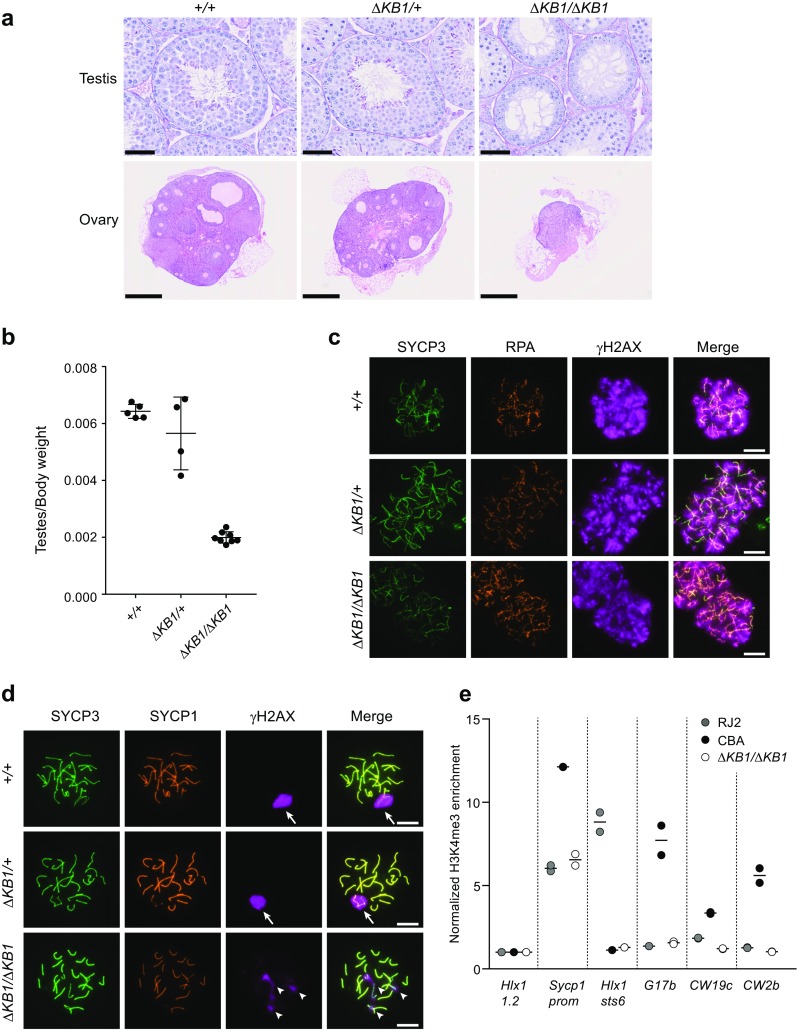
*Prdm9* ^*ΔKB1/ΔKB1*^ gonads show developmental defects associated with synaptic defects at prophase I. **a** Representative images of Periodic acid-Schiff-stained paraffin sections of 10-week-old *Prdm9* ^*+/+*^, *Prdm9* ^*ΔKB1/+*^, and *Prdm9* ^*ΔKB1/ΔKB1*^ testes (*scale bars* = 50 μm) and hematoxylin-eosin-stained paraffin sections of 10-week-old *Prdm9* ^*+/+*^, *Prdm9* ^*ΔKB1/+*^, and *Prdm9* ^*ΔKB1/ΔKB1*^ ovaries. Two mice for each genotype (except for *Prdm9* ^*ΔKB1/+*^ ovaries) were analyzed. *Scale bars* = 500 μm. **b** Testes/body weight ratio in *Prdm9* ^*+/+*^ (*n* = 5), *Prdm9* ^*ΔKB1/+*^ (*n* = 4), and *Prdm9* ^*ΔKB1/ΔKB1*^ (*n* = 8) mice. **c** Immunofluorescent analysis of RPA (DSB repair marker), SYCP3 (meiotic chromosomes axial elements marker), and γH2AX (DSB formation marker) in chromosome spreads of *Prdm9* ^*+/+*^, *Prdm9* ^*ΔKB1/+*^, and *Prdm9* ^*ΔKB1/ΔKB1*^ spermatocytes at zygonema. *Scale bars* = 10 μm. **d** Immunofluorescent detection of SYCP1 (synapsis marker), SYCP3 (meiotic chromosomes axial elements marker), and γH2AX (sex body and DSB formation marker) in chromosome spreads of *Prdm9* ^*+/+*^, *Prdm9* ^*ΔKB1/+*^, and *Prdm9* ^*ΔKB1/ΔKB1*^ spermatocytes at pachynema. *Arrows* indicate the sex body. *Arrowheads* indicate unsynapsed axes. *Scale bars* = 10 μm. **e** H3K4me3 enrichment at CBA hotspots in RJ2, CBA, and CBA *Prdm9* ^*ΔKB1/ΔKB1*^ testes. *Hlx1 1.2*, *Sycp1prom* (promoter region of the *Sycp1* gene), and *Hlx1 sts6* were used as control loci. CBA hotspots are *G17b*, *CW19c*, and *CW2b*. H3K4me3 enrichment relative to input at each locus was normalized to the *Hlx1 1.2* values. Data from two technical replicates of independent ChIP experiments (*circles*) and their average value (*horizontal bar*) are shown

We identified both oogenesis and spermatogenesis defects in *Prdm9* ^*ΔKB1*/*ΔKB1*^ mice. Histological analysis showed that ovary development was compromised with the absence of growing primary and primordial follicles in adult ovaries. Adult testes presented defects in spermatogenesis with the absence of haploid cells (both spermatids and spermatozoa) (Fig. 2a). Testis weight was also strongly reduced in *Prdm9* ^*ΔKB1*/*ΔKB1*^ males (Fig. 2b). Conversely, we did not find any significant difference in spermatogenesis and testis weight in wild-type and *Prdm9* ^*ΔKB1*/*+*^ testes. To specifically examine the progression through meiotic prophase, we analyzed spread spermatocytes and monitored DSB formation, DSB repair, and synapsis by detection of phosphorylated histone H2AX (γH2AX), RPA, and SYCP1, respectively. DSBs were formed abundantly in *Prdm9* ^*ΔKB1*/*ΔKB1*^ leptotene and zygotene spermatocytes (Figs. 1c and 2c). However, spermatocytes never progressed to the normal pachytene stage where all autosomes are fully synapsed and sex chromosomes are incorporated into a γH2AX-rich chromatin domain, called the sex body (arrows in Fig. 2d). Instead, at the most advanced stage, we detected one or several asynaptic autosomes (arrowheads) in addition to apparently fully synapsed chromosomes with fully condensed axes that are characteristic of the pachytene stage (Fig. 2d). High γH2AX levels were present on asynaptic autosomes and sex body formation was defective in these cells (Fig. 2d). We could still observe RPA foci at this pachytene-like stage in *Prdm9* ^*ΔKB1*/*ΔKB1*^ spermatocytes which also were weakly stained with testis-specific histone H1t (Supplementary Fig. 3). In wild type, at a comparable stage of progression in meiotic prophase, spermatocytes positive for H1t display a reduced number of RPA foci (Supplementary Fig. 3). Thus, *Prdm9* ^*ΔKB1*/*ΔKB1*^ spermatocytes maintain a high level of RPA foci due to DSB repair defects and/or arrest in meiotic prophase. These phenotypes are similar to those observed in *Prdm9* ^−/−^ mice (Hayashi et al. 2005; Sun et al. 2015).

The role of PRDM9 in specifying meiotic DSB sites involves its binding to DNA motifs and is associated with methylation of H3K4 and H3K36 on adjacent nucleosomes (Baker et al. 2014; Brick et al. 2012; Buard et al. 2009; Grey et al. 2011, 2017; Powers et al. 2016; Smagulova et al. 2011), leading to DSB formation in the immediate vicinity of PRDM9-bound sites (Lange et al. 2016). To analyze if PRDM9 methyltransferase activity is impaired in *Prdm9* ^*ΔKB1*/*ΔKB1*^ mice, we monitored H3K4me3 enrichment at hotspots known to be specified by the *Prdm9* CBA allele (*G17b*, *CW19c*, and *CW2b*) on the basis of the enrichment in H3K4me3 and DMC1 from previous studies (Baker et al. 2015a; Smagulova et al. 2016). Here, we found H3K4me3 enrichment at the *G17b*, *CW19c*, and *CW2b* hotspots in testis chromatin from CBA but not from *Prdm9* ^*ΔKB1*/*ΔKB1*^ mice (Fig. 2e).

We conclude that the KRAB domain is important for PRDM9 activity and that the phenotypes observed in *Prdm9* ^*ΔKB1*/*ΔKB1*^ spermatocytes could be due to the lower level of the truncated protein and/or to a loss of its specific activities such as methyltransferase, DNA-binding, and/or protein interaction. This may lead to the absence of DSB formation at the potential binding sites for PRDM9. As γH2AX and RPA foci are still present in *Prdm9* ^*ΔKB1*/*ΔKB1*^ spermatocytes, we hypothesize that DSBs are formed in *Prdm9* ^*ΔKB1*/*ΔKB1*^ mice, possibly near transcription regulatory sites, such as H3K4me3-enriched promoters and enhancers, as observed in *Prdm9* ^−/−^ mice (Brick et al. 2012).

### Identification of PRDM9 partners

The KRAB domain is involved in protein interactions and mediates transcriptional repression (Lupo et al. 2013). The domain present in PRDM9 is more closely related to that of SSX proteins than of canonical KRAB-C2H2 zinc finger proteins (Birtle and Ponting 2006). Notably, the atypical KRAB domains of SSX proteins harbor substitutions at KRAB A domain residues that are required for transcriptional repression and for the interaction with the transcriptional regulator TRIM28 (Lim et al. 1998). Accordingly, in vitro, human PRDM9 KRAB does not interact with TRIM28 (Patel et al. 2016). By Y2H assay, we found that TRIM28 did not interact with mouse PRDM9 KRAB (Supplementary Fig. 4a, b), whereas it interacted with the canonical KRAB domain of KOX1, as previously shown (Peng et al. 2000).

**Fig. 3:**
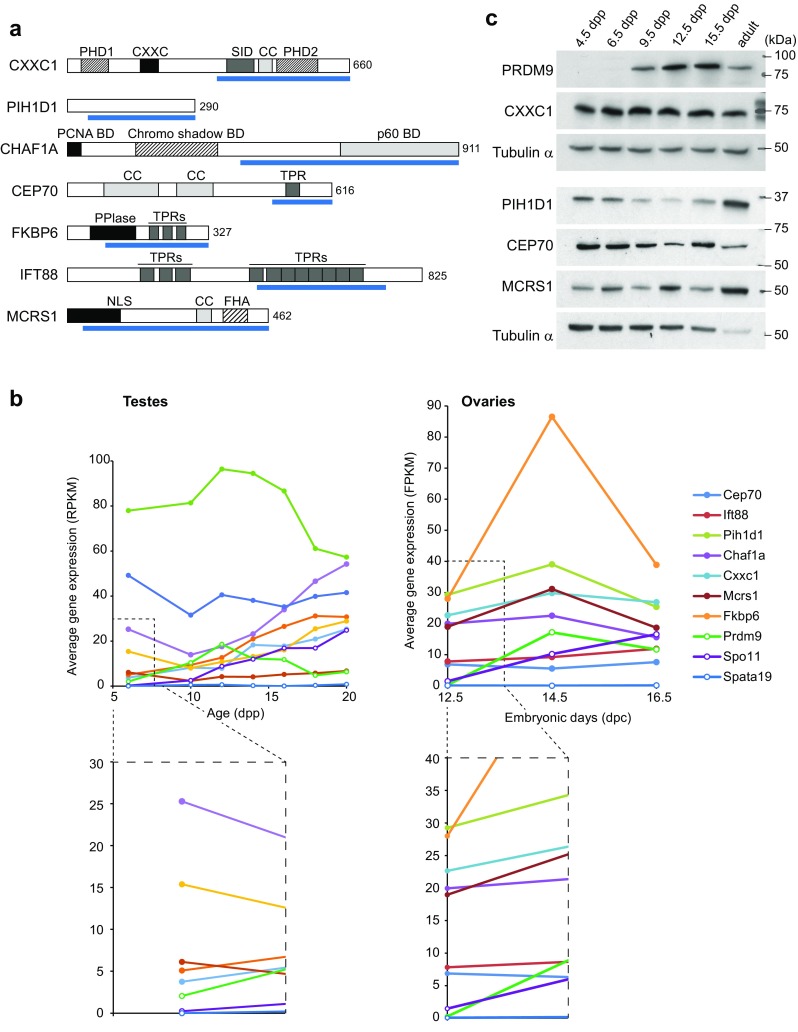
Identification of interacting partners of PRDM9 by Y2H screening. **a** Schematic representation of the domain structure of seven PRDM9-interacting proteins identified by Y2H screening. The prey fragments identified in the screens are shown as *blue bars* underneath each protein. The length (number of amino acid residues) of each full-length protein is indicated on the *right*. **b** RNA expression of the PRDM9-interacting proteins in mouse testes (*left*) and ovaries (*right*) during meiosis. Data were extracted from RNA-seq data in Margolin et al. (2014) and Soh et al. (2015)). The average gene expression is shown in reads per kilobase per million of reads (RPKM) and fragments per kilobase of transcript per million mapped read (FKPM) for testes and ovaries, respectively. Zoomed-in views of the *dashed line boxes* are shown below. *Spo11* and *Spata19* expression were used as positive and negative controls, respectively. **c** Western blot analysis of PRDM9, CXXC1, PIH1D1, CEP70, and MCRS1 expression in whole testis extracts from 4.5, 6.5, 9.5, 12.5, and 15.5 dpp and adult mice. Each lane was loaded with 50 μg of testis protein extracts. Tubulin α was used as internal control

To identify potential partners, we performed two independent Y2H screens with mouse and human PRDM9 (see “Materials and methods,” Supplementary Table 1). As no common protein was detected in these two screens, we assayed the interaction with PRDM9 of both mouse and human orthologues for 12 and 4 selected candidates from the mouse and human screens, respectively. This led to the conclusion that both mouse and human PRDM9 interacted with CXXC1, PIH1D1, CHAF1A, CEP70, FKBP6, IFT88, and MCRS1 from mouse and human, respectively (Fig. 3a and Supplementary Table 2). According to available mouse RNA-seq data sets (Margolin et al. 2014; Soh et al. 2015), the genes encoding these seven proteins are expressed in prepubertal testes, where the first wave of spermatogenesis takes place, as well as in embryonic ovaries, where oocytes are in meiotic prophase (Fig. 3b). Differently from *Prdm9* and *Spo11*, these genes are expressed even before meiosis entry at 12.5 days post-coitum (dpc) during oogenesis (Fig. 3b). Taking advantage of the availability of antibodies against four of these proteins (CXXC1, PIH1D1, CEP70, and MCRS1), we could demonstrate their expression in testis extracts from prepubertal mice before meiosis entry (4 and 6 days post-partum, dpp), at later stages when spermatocytes proceed into meiotic prophase (9 to 15 dpp), and in adult mice (Fig. 3c).

To identify the PRDM9 region involved in these interactions, we generated various mouse PRDM9 expression plasmids that encoded one of its main domains: the KRAB domain, a region encoded by exon 5, the SSX Repressor Domain (SSXRD) that acts as a transcription repressor in SSX proteins (Lim et al. 1998), the pre-SET and SET domains involved in methyltransferase activity (Wu et al. 2013), and the linker sequence between the SET domain and the zinc finger array (Supplementary Fig. 5). The zinc finger array was not included because the bait used for the Y2H screening with the mouse cDNA library was PRDM9 without the zinc finger array. This analysis revealed that the KRAB domain was involved in all interactions (Table 1). In addition, the region encoded by exon 5 was involved in interactions with PIH1D1 and CHAF1A, whereas SSXRD weakly interacted with CXXC1 and CHAF1A. Therefore, the KRAB domain of PRDM9 is potentially involved in mediating interactions with various partners.

**Table 1:** Mapping of interacting domains of PRDM9 by yeast two-hybrid assay

			Gal4 BD-PRDM9						
			FL	KRAB^a^	Exon 5	SSXRD^a^	Pre-SET	PR/SET	Linker
								Y357F^a^	
Gal4 AD	CXXC1	348–660	+++	+++	−	+	−	−	−
	MCRS1	FL	+++	++	−	−	−	−	−
	PIH1D1	42–290	+++	+++	+++	−	−	−	−
	CHAF1A	397–911	++	+	++	+	−	−	−
	CEP70	FL	++	++	−	−	−	−	−
	FKBP6	85–327	++	+++	−	−	−	−	−
	IFT88	FL	++	++	−	−	−	−	−
	Empty vector		−	−	−	−	−	−	−

Gal4 BD was fused to mouse full-length PRDM9 (FL) or domains shown in Supplementary Fig. 5. Gal4 AD was fused to full-length (FL) or fragments (indicated in positions of amino acid residues) of mouse orthologues of seven proteins

− no growth on −LWH, + few colonies on −LWH, ++ normal growth on −LWH, +++ growth on both −LWH− and –LWHAd

^a^Amino-triazole was added to −LWH at 5 mM when constructs with self-activation activity were tested

### CXXC1, the mouse orthologue of yeast Spp1, interacts with PRDM9 through its SET1-interacting domain

**Fig. 4:**
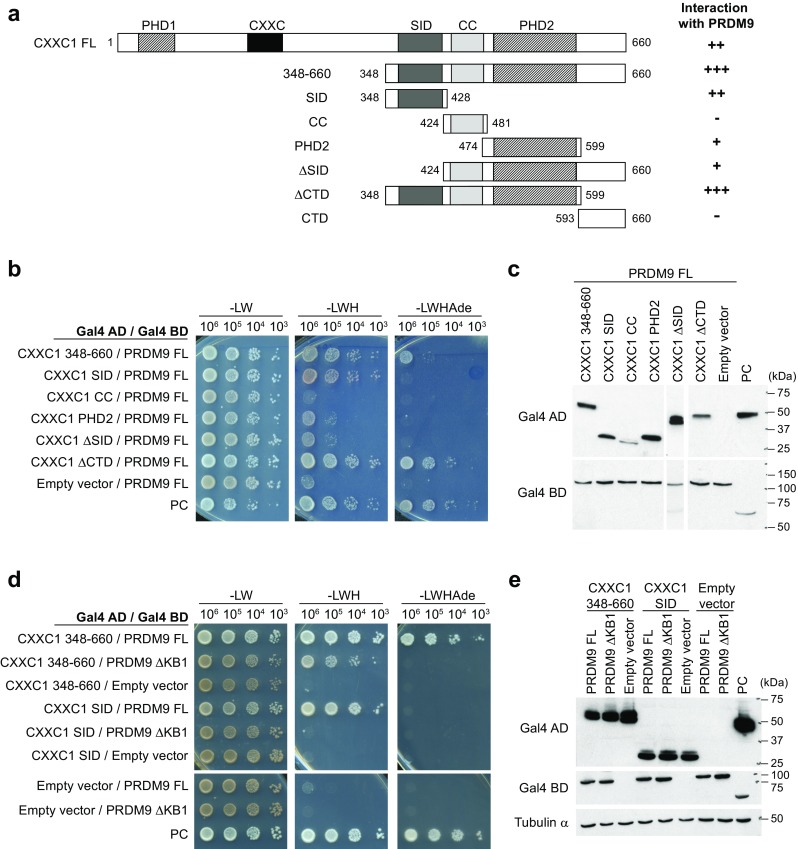
The SET1-interacting domain (*SID*) of CXXC1 mediates the interaction with PRDM9. **a** Schematic representation of the mouse CXXC1 constructs used for domain mapping. *Numbers* correspond to the position of the amino acid residues from the N-terminus. *PHD1* plant homeodomain 1, *CXXC* CXXC-type zinc finger, *SID* SET1-interacting domain, *CC* coiled-coil, *PHD2* plant homeodomain 2, *CTD* C-terminal domain. Interactions with mouse PRDM9 detected by Y2H assay are summarized on the *right*. Interactions with full-length CXXC1 (*FL*) and CTD were examined by streaking on selective media (data not shown). **b** Mapping of the PRDM9-interacting domains in CXXC1 by Y2H assay using truncated CXXC1 proteins. **c** Detection of Gal4 AD-CXXC1 fusion proteins by western blotting in yeast extracts (*upper panels*). Full-length Gal4 BD-PRDM9 fusion protein, which is expected to be expressed at similar levels in the diploids tested here, was used as loading control (*lower panels*). **d** Y2H assay to test interactions between the indicated truncated CXXC1 proteins and PRDM9ΔKB1. **e** Detection of Gal4 AD-CXXC1 and Gal4 BD-PRDM9 fusion proteins by western blotting of yeast extracts. Tubulin α was used as loading control. **b**–**e** *PC* positive control

We then investigated in more details the interaction between PRDM9 and CXXC1, because Spp1, the yeast orthologue of CXXC1, plays a major role in the localization of meiotic DSBs in *S. cerevisiae* (Acquaviva et al. 2013; Sommermeyer et al. 2013). By mapping the CXXC1 domains involved in the interaction with PRDM9 (Fig. 4a–c),we showed a robust interaction involving the SET1-interacting domain (SID) (Butler et al. 2008) and a weak interaction mediated by PHD2, a putative PHD with a poor match to the consensus sequence (Voo et al. 2000) (Supplementary Fig. 6a). Interestingly, these two domains are also important for CXXC1 interaction with the SETD1A and SETD1B complexes (Butler et al. 2008). We then showed that the deletion of the KRAB domain in PRDM9ΔKB1 strongly reduced the interaction with CXXC1C-terminal region (348–660) and abolished the interaction with CXXC1 SID (Fig. 4d, e).

### The interaction between CXXC1 and IHO1 suggests a role for CXXC1 in loop/axis interaction

**Fig. 5:**
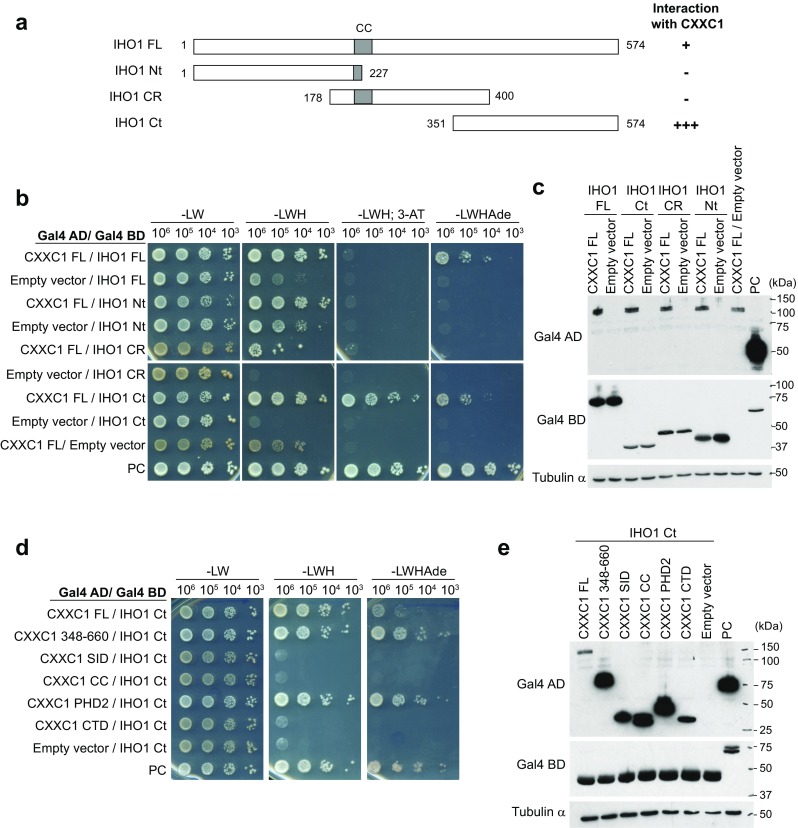
CXXC1 interacts with IHO1. **a** Schematic representation of the mouse IHO1 constructs used for testing the interaction with mouse CXXC1. *CC* coiled-coil, *CR* central region, *Ct* C-terminus, *FL* full length, *Nt* N-terminus *Numbers* correspond to the positions of amino acid residues from the N-terminus. **b** Y2H assay to test the interaction between full-length (*FL*) CXXC1 and full-length or truncated IHO1 proteins (see **a**). **c** Detection of Gal4 AD-CXXC1 and Gal4 BD-IHO1 fusion proteins by western blotting of yeast extracts. Tubulin α was used as loading control. **d** Mapping of the IHO1-interacting domain of CXXC1 by Y2H assay with truncated CXXC1 proteins and IHO1 Ct. **e** Detection of Gal4 AD-CXXC1 and Gal4 BD-IHO1 Ct fusion proteins by western blotting of yeast extracts. Tubulin α was used as loading control

**Fig. 6:**
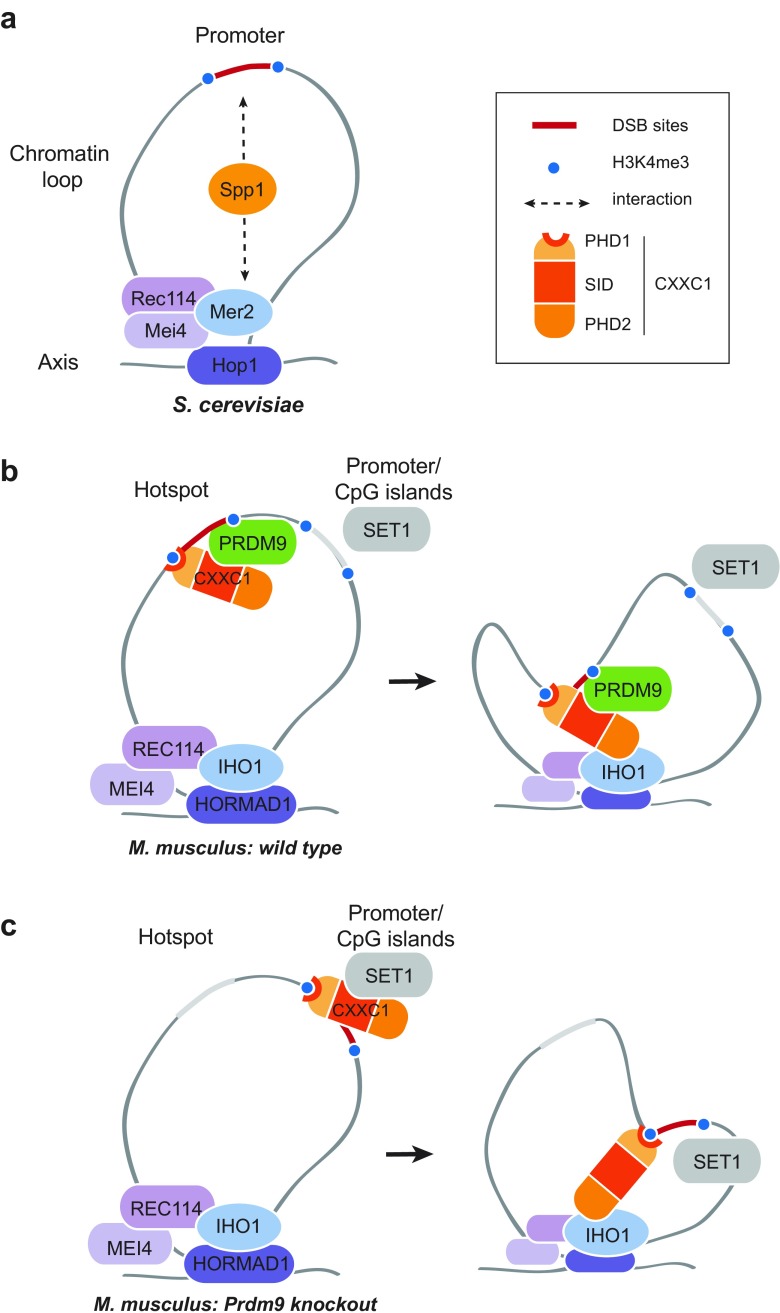
A model of the meiotic function of PRDM9 and CXXC1 in mouse meiosis. **a** In *S. cerevisiae*, Spp1 links hotspots to the Rec114-Mei4-Mer2 (*RMM*) complex through interactions with H3K4me2/me3 and Mer2 during meiosis (Acquaviva et al. 2013; Sommermeyer et al. 2013)*.* The RMM complex axis localization is Hop1-dependent (Blat et al. 2002; Panizza et al. 2011). In this panel and in the following ones, only one sister chromatid is drawn. The other sister chromatid may or not be associated and involved in these interactions. **b** At leptonema in wild-type mouse meiosis, CXXC1 is not associated with SET1 but interacts with the KRAB domain of PRDM9 through its SID. In addition, CXXC1 interacts with H3K4me3 deposited by PRDM9 via PHD1 and can interact with IHO1 via PHD2. These interactions facilitate the recruitment of hotspots to the DSB machinery on chromosomal axes. **c** In *Prdm9* knockout mice, CXXC1 could be associated with promoters/CpG islands similarly to the localization reported in somatic cells (Thomson et al. 2010). This association may involve interaction with SET1, H3K4me3, and DNA-binding through the CXXC zinc finger. CXXC1 and IHO1 interact leading to DSB formation near promoter/CpG islands (Brick et al. 2012)

*S. cerevisiae* Spp1 interacts with Mer2 and mediates indirect interactions between the chromosome axis to which Mer2 is associated and DSB sites enriched for H3K4me3, where Spp1 can bind through its PHD finger (Acquaviva et al. 2013; Sommermeyer et al. 2013). The mouse IHO1 protein shares many properties with yeast Mer2 (Stanzione et al. 2016): they are both localized on the chromosome axis, are required for MEI4/REC114 axis association and for meiotic DSB formation, and interact with REC114 (Fig. 6a). Despite the low sequence similarity, IHO1 is a Mer2 homologue (HM Bourbon, personal communication) and, therefore, might interact with CXXC1. Indeed, we detected a strong interaction between CXXC1 and the C-terminal domain of IHO1 (Fig. 5a–c). We also showed that the PHD2 of CXXC1 was sufficient for this interaction (Fig. 5d, e). As the Spp1-Mer2 interaction also involves the C-terminal region of Spp1 (Acquaviva et al. 2013) (Supplementary Fig. 6b), we searched for similarity between CXXC1 and Spp1 C-terminal regions and found four conserved residues in the PHD2 domain of CXXC1 (Butler et al. 2008) (Supplementary Fig. 6c).

However, we could not observe significant interactions between PRDM9, CXXC1, and IHO1 by co-immunoprecipitation of mouse testis extracts using various approaches. It is possible that these interactions cannot be detected in the high salt conditions required for PRDM9 extraction. Alternatively, they could be transient and/or involve only a fraction of nuclear proteins. Thus, on the basis of the interaction detected by the Y2H assays, we propose that CXXC1 may provide a link between PRDM9 sites and the chromosome axis (Fig. 6b). PRDM9 binding to its target genomic sites is associated with a local H3K4me3 enrichment on adjacent nucleosomes, predicted to be catalyzed by PRDM9 methyltransferase activity. We propose that CXXC1 is associated with these sites through the combined interaction with PRDM9 and H3K4me3, because the PHD1 of CXXC1 binds to methylated H3K4 (Eberl et al. 2013). The association of CXXC1 with IHO1, which interacts with REC114, will thus bring in proximity DSB sites and components essential for DSB formation (the potential IHO1-REC114-MEI4 complex). Whether this process is driven by diffusion and stabilization of interactions or by an active mechanism such as loop extrusion remains to be determined. SPO11-TOPOVIBL, the catalytic complex for DSB formation (Robert et al. 2016), may be initially associated with PRDM9 or with the IHO1-REC114-MEI4 complex. In any case, DSBs will be induced within the chromosome axis. We further hypothesize that in this context, CXXC1 does not interact with the SET1-COMPASS complex near transcription start sites (TSS), which could result from limiting CXX1. Our observation that the interaction domain of CXXC1 with PRDM9 overlaps the SID of CXXC1 (Fig. 4) suggests a competition between SET1 and PRDM9 for interaction with CXXC1 and therefore the formation of two alternative complexes. Consequently, CXXC1 may be titrated away from SET1 complex by PRDM9 and no DSB activity induced near TSS in the presence of PRDM9 (Fig. 6b). This competition may also explain phenotypes associated with changes in *Prdm9* gene dosage and its partial haplo-insufficiency (Baker et al. 2015b; Flachs et al. 2012). As the CXXC1 interaction domain with IHO1 is distinct from the SID (Fig. S6), one could envision the formation of a complex involving PRDM9, CXXC1, and IHO1. In the absence of PRDM9, CXXC1 might interact with loci enriched in H3K4me3 through its PHD1, as part, or not, of the SET1-COMPASS complex and with IHO1 (Fig. 6c). CXXC1 is also a DNA-binding protein with affinity for non-methylated CpGs (Lee et al. 2001) and is enriched at CpG islands (Thomson et al. 2010). This activity could contribute to stabilize its interaction in particular at CpG islands, whether these are enriched for H3K4me3 or not, and thus together or independently from its interaction with H3K4me3 and SET1-COMPASS. This would result in DSB formation at H3K4me3-enriched sites, including many promoters and at enhancers, as observed in *Prdm9* ^−/−^ mice (Brick et al. 2012) but also at CpG islands whether enriched for H3K4me3 or not as observed in dogs where *Prdm9* is a pseudogene (Auton et al. 2013).

## Materials and methods

### Mice

A 26-bp deletion in the *Prdm9* gene was generated by CRISPR/Cas9-mediated mutagenesis to produce the *Prdm9* ^*ΔKB1*^ allele. Cas9 mRNA and a single-guide RNA targeting the 5′ site of *Prdm9* (5′-TGAACACCAACAAGCTGGA-3′) were injected in B6 × CBA F1 zygotes. A founder mouse with the *Prdm9* ^*ΔKB1*^ allele was backcrossed to B6. This mutation is associated with the CBA allele of *Prdm9* that is called *Prdm9* ^*Dom3*^. B6 carry the *Prdm9* ^*Dom2*^ allele. *Prdm9* knockout (*Prdm9* ^*−/−*^) (Hayashi et al. 2005), CBA/JRj (Janvier Labs), and RJ2 (Grey et al. 2011) and B6 mice were used as controls. All experiments were carried out according to the CNRS guidelines and approved by the ethics committee on live animals (project CE-LR-0812 and 1295).

### Preparation of testis protein extracts

Adult or juvenile mouse testes were homogenized in extraction buffer (50 mM HEPES-Na pH 7.9, 400 mM NaCl, 1% Triton X, 4 mM DTT, proteinase inhibitor cocktail; Roche). After incubation at 4 °C for 30 min, samples were sonicated at high power for five cycles (30 s on, 30 s off) with a Bioruptor^®^ Standard apparatus (Diagenode). The soluble fraction was recovered by centrifugation at 14,500 rpm for 15 min. Protein concentration was determined with the Bio-Rad Protein Assay (Bio-Rad).

### Chromatin immunoprecipitation and quantitative polymerase chain reaction

Four testes from 12- or 13-dpp mice were cross-linked with 1% formaldehyde/PBS for 10 min, followed by incubation with 250 mM glycine at room temperature for 5 min. After washing in PBS, tubules were homogenized in a douncer. A single cell suspension was obtained by filtration with a 40-μm cell strainer. Cell lysis and immunoprecipitation were performed as described in Houles et al. (2015) with the following modifications: cells were lysed in 600 μl lysis buffer containing 1% SDS; sonication was carried out with a Bioruptor^®^ Standard apparatus (Diagenode) at high power for four series of 10 cycles (30 s on, 30 s off); and chromatin was 10-fold diluted in dilution buffer (5 mM Tris pH 8.0, 140 mM NaCl, 0.5% Triton X-100, 0.05% sodium deoxycholate, 0.5 mM EGTA). For immunoprecipitation, 1 ml of chromatin suspension was incubated with 20 μl of Dynabeads^®^ Protein A (Thermo Fisher Scientific) pre-incubated with 3 μg of rabbit anti-histone H3 tri-methyl K4 antibody (Abcam, ab8580).

Quantitative polymerase chain reaction (qPCR) was performed with FastStart SYBR Green Master (Roche) on a LightCycler^®^480 Instrument (Roche) and the following primers: G17b-F, 5′-CATGGACATGGAGACCTAACTG-3′; G17b-R, 5′-TCAGTGGAAGCTCAGAAAATGA-3′; CW19c-F, 5′-ACACATCTCTAAGATTATAGCTCAGCA-3′; CW19c-R, 5′-CAAAGAACATGTAGTAAACAATGAGGA-3′; CWb-F, 5′-CCTTAGGTATCCTATTGCACTGCT-3′; and CW2b-R, 5′-GGATGGGGTTGAACTAGCTG-3′. Primers for the *Hlx1 1.2*, *Sycp1prom*, and *Hlx1 sts6* sites have been reported previously (Buard et al. 2009). The *CW19c* and *CW2b* hotspots are located on chromosome 19 (GRCm38 coordinates; 19: 41,506,651–41,509,050) and 2 (2: 146,281,159–146,283,855), respectively. These sites were selected from strong peaks in DMC1 chromatin immunoprecipitation (ChIP)-seq data for the C3H mouse strain (Smagulova et al. 2016), and their H3K4me3 enrichment was confirmed in the Watkins Star Line B (WSB) strain (Baker et al. 2015a). As the CBA, C3H, and WSB mouse strains have the same *Prdm9* ^*Dom3*^ allele, hotspot locations are expected to be conserved in these three strains. *G17b* (17: 78,648,623–78,652,485) was originally identified as a B6 hotspot (Brick et al. 2012), and then DMC1 and H3K4me3 enrichment at this site was confirmed also in the C3H and WSB strains, respectively (Baker et al. 2015a; Smagulova et al. 2016). Amplification data were analyzed with the LightCycler^®^ 480 SW 1.5.1 software (Roche).

### Spermatocyte chromosome spreads and immunofluorescence

Spermatocyte chromosome spreads were prepared from 2- to 3-month-old mice by the dry-down method (Peters et al. 1997). Spreads were incubated at room temperature overnight with the following primary antibodies and dilutions: rabbit anti-PRDM9 polyclonal, 1:50 (Grey et al. 2017); guinea pig anti-SYCP3 polyclonal, 1:1000 (Grey et al. 2009); mouse anti-γH2AX monoclonal (Millipore, 05-636), 1:10,000; rabbit anti-RPA 30 kDa subunit polyclonal (gift from R. Knippers), 1:1000; guinea pig anti-histone H1t polyclonal (gift from M.A. Handel), 1:500; and rabbit anti-SYCP1 polyclonal (Abcam, ab15090), 1:400. Signals were visualized by incubation with goat anti-rabbit IgG Alexa Fluor 555 (Molecular Probes, A-21428), goat anti-guinea pig IgG Alexa Fluor 488 (Molecular Probes, A-11073), or anti-mouse IgG Alexa Flour 647 (Molecular Probes, A-31571) secondary antibodies at 37 °C for 45 min. After DAPI staining, slides were mounted using ProLong^®^ Gold (Thermo Fisher Scientific). Digital images were captured with a complementary metal–oxide–semiconductor (CMOS) camera (ORCA-Flash4.0 .LT, Hamamatsu) attached to a Zeiss Axio imager 2 microscope (Zeiss). After data acquisition with the ZEN imaging software (Zeiss), images were processed with OMERO (OME). Spreads from two mice of each genotype were analyzed for each antibody. PRDM9 signal was quantified using the CellProfiler 2.2.0 software. A CellProfiler pipeline was designed for quantification of PRDM9 in regions of interest (ROIs): DAPI-positive regions of early and late leptotene nuclei. The PRDM9 background intensity was measured outside the ROI. Then, the PRDM9 signal total intensity of the ROI was calculated and corrected for background, by subtracting the product of the measured mean background intensity by the ROI area.

### Histological analysis

Testes and ovaries from 9- to 10-week-old mice were fixed in Bouin’s solution (Sigma) at room temperature overnight or for 5 h, respectively. After dehydration and embedding in paraffin, 3-μm sections were prepared and stained with hematoxylin and eosin or with Periodic acid-Schiff. Image processing and analysis were carried out with the NDP.view2 software (Hamamatsu).

### Yeast two-hybrid screens

The human Y2H screen was performed by Hybrigenics Services, using the full-length human *Prdm9* allele A cloned in the pB27 (LexA BD fusion) or pB66 (Gal4 BD fusion) vector, as baits. The prey library was derived from human adult testis cDNAs. In total, 119 million (pB27-PRDM9) and 90.4 million (pB66-PRDM9) of interactions were examined, and 23 and 358 clones were processed, respectively. The mouse Y2H screen was performed by using mouse PRDM9 from *Mus musculus domesticus* without the zinc finger array (ΔZF, amino acids 2–512) cloned into pAS2-1 (Gal4 BD fusion) as bait. Mouse testis cDNAs were obtained from testis RNAs of 13- and 16-dpp mice and cloned in pGADT7 (Gal4 AD fusion) by using the BD Matchmaker Construction and Screening Kit (Clontech). In total, 170,000 interactions were examined, and 1115 positive clones were processed. cDNA sequences of positive clones were verified by PCR with the following primers: GADT7-5′5′-CTATTCGATGATGAAGATACCCCACCAAACC-3′ and GADT7-3′5′-GTGAACTTGCGGGGTTTTTCAGTATCTACGATT-3′. Among the 99 and 15 genes identified in the human and mouse screens, respectively (Supplementary Table 1), 16 candidates (4 and 12, respectively) with the strongest and specific interactions were tested again by Y2H assay with both human (PRDM9 A allele) and mouse (PRDM9^Dom2^ allele) full length or truncated PRDM9 cDNA (Supplementary Table 2). Seven proteins from mouse and human, CXXC1, PIH1D1, CHAF1A, FKBP6, and MCRS1 (initially identified in the mouse screen) and CEP70 and IFT88 (initially identified in the human screen), showed interaction with PRDM9 from mouse and human, respectively.

### Yeast two-hybrid assay

Most plasmids used in Y2H assays were cloned in the pGADGH (Gal4 AD fusion) or pAS2-1 (Gal4 BD fusion) vector modified for the Gateway® Gene Cloning Technology (Invitrogen); pGADT7 clones from the mouse cDNA library were also used as prey constructs. Y187 and AH109 yeast haploid strains were transformed with Gal4 AD and BD fusion plasmids, respectively, by using a standard lithium acetate method. Yeast diploid clones were obtained by mating each Gal4 AD and Gal4 BD clone together on an YPD plate at 30 °C and then streaking on SD medium lacking leucine and tryptophan (−LW). Protein interactions were examined by streaking purified diploid clones on −LW, −LW lacking also histidine (−LWH), and −LW lacking also histidine and adenine (−LWHAd). For clones showing self-activation, 5 mM 3-amino-1,2,4-triazol (3-AT) was added to −LWH plates. Interactions between Gal4 AD− and BD fusion proteins were evaluated based on cell growth after 3 days at 30 °C. At least two clones were examined for all constructs. Cell growth of several clones was also examined by spotting serial dilutions of diploid cells (10^6^, 10^5^, 10^4^, 10^3^ cells/spot) on −LW, −LWH, and −LWHAd plates. A diploid clone expressing Gal4 AD-REC114 and Gal4 BD-MEI4 was used as a control for positive interaction (referred to as positive control (PC)) (Kumar et al. 2010).

### Protein extraction from yeast cells

Diploid yeast cells were pre-cultured in 5 ml of SD liquid medium lacking leucine and tryptophan at 30 °C overnight. Cells were then diluted to OD_600_ ∼0.25 in 5 ml of YPD medium. After incubation at 30 °C for 4–6 h, cells were collected at OD_600_ ∼1.0. Cell pellets were washed twice in cold water and then suspended in 200 μl water. Cell lysis was performed by adding 200 μl of 0.2 M NaOH and incubating at room temperature for 5 min. After centrifugation at 12,000 rpm for 1 min, pellets were dissolved in Laemmli buffer and then incubated at 95 °C for 5 min. Protein extracts, equivalent to 0.5 OD_600_ units, were used for immunoblotting. Extracts from diploid cells expressing Gal4 AD-REC114 and Gal4 BD-MEI4 were loaded as positive control.

### Immunoblotting

Protein extracts were separated on 7.5 or 10% Mini-PROTEIN TGX Precast Gels (Bio-Rad) and then transferred onto nitrocellulose membranes with the Trans-Blot Turbo Transfer System (Bio-Rad). The following primary antibodies and dilutions were used: rabbit anti-PRDM9 polyclonal (Grey et al. 2017), 1:500; rabbit anti-CTCF monoclonal (Cell Signaling Technology, D31H2), 1:2000; rabbit anti-CXXC1 polyclonal (Millipore, ABE211), 1:5000; rat anti-tubulin α monoclonal (Abcam, ab6161), 1:3000; rabbit anti-PIH1D1 polyclonal (Proteintech, AP19427), 1:5000; rabbit anti-CEP70 polyclonal (Abnova, PAB17658), 1:1000; mouse anti-MCRS1 polyclonal (Abnova, H00010445-B01P), 1:250; anti-Gal4 AD (Millipore, 06-283), 1:3000; and anti-Gal4 BD (Sigma, G3042), 1:2000. Signals were detected with the horseradish peroxidase (HRP)-conjugated secondary antibodies, donkey anti-rabbit IgG HRP (Jackson ImmunoResearch Laboratories), goat anti-rat IgG HRP (GE Healthcare), and sheep anti-mouse IgG HRP (GE Healthcare), and then visualized with SuperSignal West Pico Chemiluminescent Substrate (Thermo Fisher Scientific). ImageJ 1.49m was used to quantify signal intensities of western blot images captured by ChemiDoc™ XRS+ Imager with the Image Lab 5.1 software.

### Protein sequence alignment

Multiple protein sequence alignments were carried out using the T-Coffee Multiple Alignment program (http://www.ebi.ac.uk/Tools/msa/tcoffee) and the BLOSUM matrix. Alignment results were visualized with BoxShade (http://www.ch.embnet.org/software/BOX_form.html).

## Electronic supplementary material

Supplementary Fig. 1 Generation of the *Prdm9* ^*ΔKB1*^ allele by CRISPR-Cas9 mediated mutagenesis. **a**. Protein sequence alignment of the KRAB domains from human PRDM9 (Q9NQV7), mouse PRDM9 (Q96EQ9), proteins of the human SSX family (SSX1: Q16384, SSX2: Q16385, SSX3: Q99909, SSX4: O60224, SSX5: O60225), and human KOX1 (P21506) (Lim et al. 1998). Hs: *Homo sapiens*, Mm: *Mus musculus domesticus.* Dashes indicate gaps. Amino acid residues the substitution of which in KOX1 KRAB abolishes the interaction with TRIM28 are indicated by red arrows (Margolin et al. 1994; Moosmann et al. 1996). *Prdm9* ^*ΔKB1*^ translation is predicted to start at the methionine at position 43 (black arrowhead). **b** Targeting the translation start site of *Prdm9* by single-guide RNA (sgRNA). The predicted cleavage site is indicated by a red arrow. The protospacer adjacent motif (PAM) required for the cleavage is underlined. **c**. The 26 bp deletion associated with the *Prdm9* ^*ΔKB1*^ allele. Sequencing data for the wild type *Prdm9* ^*B6*^ and *Prdm9* ^*ΔKB1*^ alleles are shown. **d** Predicted protein sequences for wild type B6 PRDM9 and CBAPRDM9ΔKB1. Red letters indicate the first methionine of each sequence. Odd-numbered exons are shown in boxes; the exon-intron structure of the gene is according to the GenBank entry NM_144809.2. (AI 23615 kb)

Supplementary Fig. 2 Quantification of PRDM9 signals on spermatocyte chromosome spreads. **a** Representative images of PRDM9-stained nuclei of *Prdm9* ^*+/+*^, *Prdm9* ^*ΔKB1/+*^, *Prdm9* ^*ΔKB1/ΔKB1*^, and *Prdm9* ^*−/−*^ spermatocytes, used for quantification of PRDM9 signals. Both strongly and weakly stained nuclei for PRDM9 are shown for each genotype. Leptotene nuclei were selected based on the SYCP3 staining pattern in chromosome spreads prepared from 10-week-old mice. *Scale bars* = 10 μm. **b.** Quantification of PRDM9 signal intensities in leptotene nuclei of *Prdm9* ^*+/+*^, *Prdm9* ^*ΔKB1/+*^, *Prdm9* ^*ΔKB1/ΔKB1*^, and *Prdm9* ^*−/−*^ spermatocytes. The total intensities (in arbitrary units, a.u.) of PRDM9 in each nucleus were normalized by background signals (see “Materials and methods”). Leptotene nuclei were classified as early or late leptonema, according to the SYCP3 staining patterns. The total number of early and late leptotene nuclei scored was 47 and 39 for *Prdm9* ^*+/+*^, 33 and 35 for *Prdm9* ^*ΔKB1/+*^, 33 and 33 for *Prdm9* ^*ΔKB1/ΔKB1*^, and 49 and 46 for *Prdm9* ^*−/−*^. The vertical and horizontal bars represent standard deviations (SD) and mean intensities, respectively. Values of mean ± SD are: *Prdm9* ^*+/+*^ (6857 ± 2830), *Prdm9* ^*ΔKB1/+*^ (6130 ± 2731), *Prdm9* ^*ΔKB1/ΔKB1*^ (5296 ± 2002), and *Prdm9* ^*−/−*^ (2775 ± 835) at early leptonema, and *Prdm9* ^*+/+*^ (7416 ± 3220), *Prdm9* ^*ΔKB1/+*^ (6569 ± 2761), *Prdm9* ^*ΔKB1/ΔKB1*^ (4353 ± 1799), and *Prdm9* ^*−/−*^ (3082 ± 544) at late leptonema. The Mann-Whitney test was performed to assess statistical significance (*p* values are shown above the graph). (AI 11011 kb)

Supplementary Fig. 3 *Prdm9* ^*ΔKB1/ΔKB1*^ spermatocytes at the most advanced stages are positive for both H1t and RPA. Immunofluorescence analysis of histone H1t (mid-pachytene marker), RPA (DSB repair marker), and γH2AX (DSB formation marker) in chromosome spreads of *Prdm9* ^*+/+*^ and *Prdm9* ^*ΔKB1/ΔKB1*^ spermatocytes, prepared from 10-week-old mice. Wild type nuclei at zygonema to mid/late-pachynema (left panel) and *Prdm9* ^*ΔKB1/ΔKB1*^ nuclei at most advanced stages (right panel) are presented. Scale bars = 10 μm. (AI 34915 kb)

Supplementary Fig. 4 The atypical KRAB domain of PRDM9 does not interact with TRIM28. **a** Y2H assay to test the interaction between the N-terminal fragment of TRIM28 (amino acid residues 1–419) and full length (FL) mouse PRDM9 or the KRAB domain of mouse PRDM9 or of human KOX1. **b** Detection of Gal4 AD-TRIM28 and Gal4 BD-PRDM9 FL, Gal4 BD-PRDM9 KRAB, or Gal4 BD-KOX1 KRAB fusion proteins by western blotting of yeast extracts. Tubulin α was used as loading control. PC, Positive Control. (AI 5803 kb)

Supplementary Fig. 5 PRDM9 domains. Schematic representation of the PRDM9 constructs used for domain mapping. Numbers correspond to the positions of amino acid residues from the N-terminus. (AI 1972 kb)

Supplementary Fig. 6 Evolutionary conservation of functional domains between mouse CXXC1 and *S. cerevisiae* Spp1. **a** Domain structure of mouse CXXC1. The domains interacting with SET1A (SET1-ID: 1415–1538) (Butler et al. 2008), PRDM9 (PRDM9-ID: 348–428 and 474–599), and IHO1 (IHO1-ID: 474–599) are shown. **b** Domain structure of *S. cerevisiae* Spp1. The domain interacting with Mer2 (Mer1-ID: 223–354) (Acquaviva et al. 2013) is shown. The positions of the plant homeodomain (PHD), Spp1 zinc finger 1/2 (SZF1/2) and the predicted DNA-binding region are based on (Murton et al. 2010). **c** Protein sequence alignment of the C-terminal region of the PHD2 domain of CXXC1 and its orthologue Spp1. Dashes indicate gaps. The four conserved residues within the conserved motif are shown below. Residues in white over black, white over grey and black over grey indicate 100, 80 and 60% conservation, respectively. The protein sequences used for the alignment were (RefSeq protein ID is between brackets): *Candida albicans* (XP_721516.1), *Komagataella phaffii* (XP_002491094.1), *Saccharomyces cerevisiae* (NP_015187.1), *Kluyveromyces lactis* (XP_453385.1), *Candida glabrata* (XP_449459.1), *Zygosaccharomyces rouxii* (XP_002497491.1), *Lachancea thermotolerans* (XP_002553172.1), *Aphis gossypii* (NP_983517.2), *Homo sapiens* (NP_055408.2), *Anopheles gambiae* (XP_318129.4), *Mus musculus* (NP_083144.1), *Danio rerio* (NP_956893.1), *Bos taurus* (NP_001019688.1), and *Canis lupus familiaris* (*XP_537342.2*). (AI 4071 kb)

Supplementary Table 1 Lists of proteins identified in the Y2H screens with PRDM9 as bait. Proteins identified in Y2H screens with human (a) and mouse (b) testis cDNA libraries. IF: in frame; OOF: out of frame; ??: not determined; n.a.: non-coding sequence. Proteins re-examined with both human and mouse cDNA clones by Y2H assays are indicated by red letters. Proteins identified in the human screen were ranked by predicted biological scores (PBS), provided by algorithms of Hybrigenics Services; A: very high confidence; B: high confidence; C: good confidence; D: moderate confidence, including potential false positives; E: interactions involving highly connected prey domains, warning of non-specific interaction; N/A: not applicable. (XLSX 41 kb)

Supplementary Table 2 Y2H assays with human and mouse orthologues of 16 proteins identified in the primary Y2H screens Interactions between PRDM9 (either human or mouse) and the 16 proteins identified in the primary Y2H screens were tested by using human and mouse orthologues. Gal4 BD was fused to human/mouse PRDM9 of full length (FL) or fragments including the amino acid indicated (∆ZFs constructs include aa 2–522 of human PRDM9 and 2–512 of mouse PRDM9). Gal4 AD was fused to both human and mouse orthologues of 16 proteins identified in the primary Y2H screens (12 proteins from the 1st mouse screen and 4 proteins from the 1st human screen). The seven proteins showing interactions for both mouse and human orthologs are highlighted in red. -: no growth on -LWH, +: few colonies on -LWH, ++: normal growth on -LWH, +++: growth on both -LWH- and -LWHAd. n.t.: not tested. PC: positive control. NC: negative control. Amino-triazol was added to -LWH at 5 mM when constructs with self-activation activity were detected (marked with *). In context where interactions were detected with all four Gal4 BD-PRDM9 constructs a control with empty vector was included. n.t.: not tested. (XLSX 17 kb)

## Abbreviations

dpp: Days post-partum
Y2H: Yeast two-hybrid
FL: Full length
